# The Need for Uniformity in the Quality of Drugs

**Published:** 1908-08-08

**Authors:** 


### Therapeutic Notes.

### THE NEED FOR UNIFORMITY IN THE QUALITY OF DRUGS.

The varying results obtained from the use of the same remedial agent have frequently been remarked by practitioners of medicine; and the difference in the activity of galenical preparations—leaving the temperament and idiocyncrasies of the patient out of the question—is capable of explanation. The quality of a prepared product varies in proportion to the varying quality of the drug from which it is prepared, and as the same galenical preparation is manufactured from widely different varieties of the same drug, it follows that the finished product, although made by the same official process, is of a variable standard. While it is impossible in the case of a large number of preparations to guarantee them to be exactly identical in strength, the importance of ensuring that they shall be as nearly approaching to uniformity as practicable cannot be too strongly urged. More than one product has lost a good reputation because of its uncertain action, which may have been due to the poor quality of the drug employed; while the danger which might follow the use of a preparation supposed to be of a definite strength, but actually twice as active as that on which the dose has been based, is obvious.

In a few cases chemical standardisation may be a way out of the difficulty, and in some instances physiological standardisation may be relied upon to ensure uniformity. But both methods have their limitations and deficiencies. Supposing the assumption, that the therapeutic activity of a drug is due to a definite chemical substance, to be correct, chemical standardisation might solve the question; but there remains the doubt that the precise condition in which that substance exists in combination with other constituents in the same drug may be a factor which influences materially its pharmacological activity. There are, in fact, very few galenical preparations which are so simple in character as to be expressed in the terms of a chemical substance. Without dwelling unnecessarily upon the deficiencies of chemical standardisation, it is unfortunate that this method of obtaining so-called uniform preparations may possibly encourage " faking " among a certain class of manufacturers who use poor qualities of drugs and raise the preparation to the necessary strength by the addition of a sufficient quantity of active ingredient. While such a preparation might conform to the required tests, it could hardly be called genuine in the true sense of the word. It is a well-known fact that while brandy actually prepared from grapes may not fulfil the necessary requirements as to purity, it is possible to prepare in the laboratory a sample of " brandy " that will come up to standard. Similarly a standard galenical preparation may be made from an inferior drug, for the possibility exists of resorting to sophistication in order to increase its alkaloidal contents. As regards physiological standardisation—however useful this method may be in certain cases—it is clear that such a system cannot be perfect so long as the animals on which the experiments are conducted are of a variable " standard." Thus, while recognising the possibilities of the chemical and physiological methods of standardisation, it is a fact that in most cases neither can be thoroughly relied upon to ensure uniformity.

The craze for cheap commodities, which is so characteristic of the present day, has also invaded the drug business. Crude drugs of good quality can be obtained just as readily as they could 20 years ago; but, unfortunately, people are not so ready to pay the price. To-day Cape aloes of fine quality is worth about 50s. per cwt., but drossy stuff can be bought for 25s. Asafœtida can be bought as low as 20s. per cwt., and as high as 120s. Genuine balsam copaiba costs double that of doubtful quality, and gum benzoin can be bought at all prices to suit the purchaser: the same remarks apply to jalap, and, in fact, to a large number of drugs. It would be unreasonable to expect to obtain the same preparation from a drug costing, say, 1s. per lb., as from one costing double the price, and it is eminently desirable that a more definite description of crude drugs should be stated in the British Pharmacopœia. In the meantime, however, preparations should be made from the best qualities of drugs obtainable, for by this means uniformity in the strength of most preparations—sufficient for all practical purposes—might be arrived at, and, with certain exceptions, standardisation of the finished product would not be necessary.

